# The Net Promoter Score (NPS) for Insight Into Client Experiences in Sexual and Reproductive Health Clinics

**DOI:** 10.9745/GHSP-D-18-00068

**Published:** 2018-10-03

**Authors:** Rebecca Koladycz, Gwendolyn Fernandez, Kate Gray, Heidi Marriott

**Affiliations:** a Independent consultant, Seattle, WA, USA.; b International Planned Parenthood Federation/Western Hemisphere Region, New York, NY, USA. Now with Keyrus, New York, NY, USA.; c International Planned Parenthood Federation, London, UK.

## Abstract

The Net Promoter Score (NPS) metric, commonly used by Fortune 500 companies to measure the customer experience, is calculated using a 0-to-10 scale to answer 1 question: “How likely is it that you would recommend [company X] to a friend or colleague?” Despite the value of this methodology as a predictor of growth and indicator of customer satisfaction in for-profit industries, uptake of the NPS has been slower in the social sector due to concerns about its applicability and acceptability in noncommercial settings, particularly among low-literacy populations. To address these concerns, we conducted a series of small-scale pilots in El Salvador, India, Kenya, and Nigeria to test different implementation approaches of the NPS in sexual and reproductive health clinics—including face-to-face interviews, a guided drop box, integration of the NPS question into an existing client exit interview, and self-administered and volunteer-assisted online surveys using tablets in clinics—and compared the traditional 0-to-10 number scale with an emoji-face scale. Findings showed that the NPS can be effectively adapted for use in low-resource health clinics among low-literacy clients using the number scale. There was no statistically significant difference in mean likeliness to recommend services when using the emoji versus numerical scales in India; however, there was a statistically significant difference when using the guided drop box approach versus face-to-face interviews. When combined with demographic and service-use questions, the NPS generated useful insights on client groups that were more or less likely to recommend the services. While providing an online survey on tablets can be an efficient methodology for implementing the NPS, self-administered approaches may be limited by a client's level of literacy or comfort with technology. For those client populations with a lower NPS, we advise using a qualitative feedback process that can elicit critical feedback to identify actions to improve their experience. Our experience with testing and implementing the NPS in SRH clinics in diverse settings suggests it is a promising approach to gaining insight into the client experience in nonprofit health care settings.

## Abstract (precis)

The NPS measures a customer's likeliness to recommend a company to a friend or colleague on a 0-to-10 scale. Pilot testing in 4 countries suggests the NPS can also be successfully used in nonprofit clinics and among low-literacy populations. Combining the NPS with client demographic and service-use data can provide a powerful tool for identifying populations for whom the client experience can be improved.

Resumen en español al final del artículo.

## BACKGROUND

In the business world, customer feedback is widely recognized as critical for providing information to improve products and services that, in turn, enable a company to increase sales and market share. Similarly, the not-for-profit sector has increasingly recognized the importance of listening to beneficiaries as a mechanism for improving the effectiveness of social programs.^1^

Within the field of reproductive health, improving the quality of services—encompassing both clinical standards and the patient experience—is a key component to increasing the use of voluntary family planning and generating demand for services.^2^ While a positive patient experience is a priority in itself, it is also important because it improves correct family planning method use, contraceptive continuation rates, and consistent use of health services.^2^ The importance of a positive experience is amplified when considering the role of word-of-mouth referrals in marketing strategies^3^ and the potential for generating demand for family planning services in countries with low modern contraceptive prevalence rates (mCPRs).

Family planning service delivery organizations, including International Planned Parenthood Federation (IPPF), have a long history of obtaining client—also known as patient or beneficiary—feedback on their experience with seeking and receiving services. Like most health care providers, we have traditionally relied on lengthy client satisfaction surveys to gather that feedback. While such client satisfaction surveys can provide useful information, our field-based partners report that these surveys are cumbersome, expensive, and often result in an overabundance of data that are difficult to analyze, interpret, and prioritize. Conversely, asking whether clients would recommend the services to others using a binary yes/no scale frequently results in more than 95% of clients reporting that they would recommend the services. In both cases, clinic staff have difficulty using such data to improve the patient experience.

Furthermore, IPPF operates across a myriad of country contexts, client populations, and service delivery settings, delivering hundreds of millions of sexual and reproductive health (SRH) services in 46,000 service delivery points located in more than 140 countries. Such a wide range of contexts makes the identification of a simple, standardized client satisfaction survey challenging.

To strengthen, streamline, and systematize feedback mechanisms on the patient experience, we began testing the use of the Net Promoter Score (NPS) in SRH clinics with low-literacy populations as an alternative approach to monitoring and improving the client experience.

## The Net Promoter Score

In 2003, Frederick F. Reichheld argued that asking a single survey question about a customer's willingness to recommend a product or service served as a strong predictor of growth in sales and revenue.^4^ In response to the question, “How likely is it that you would recommend [company X] to a friend or colleague?”, customers would rate their willingness to recommend a product or service using a 0-to-10 scale. Often, this question was followed by an open-ended question, “What is the reason for your score?”

The traditional Net Promoter Score question uses a 0-to-10 scale and asks customers “How likely is it that you would recommend [company X] to a friend or colleague?”

**FIGURE 1:**
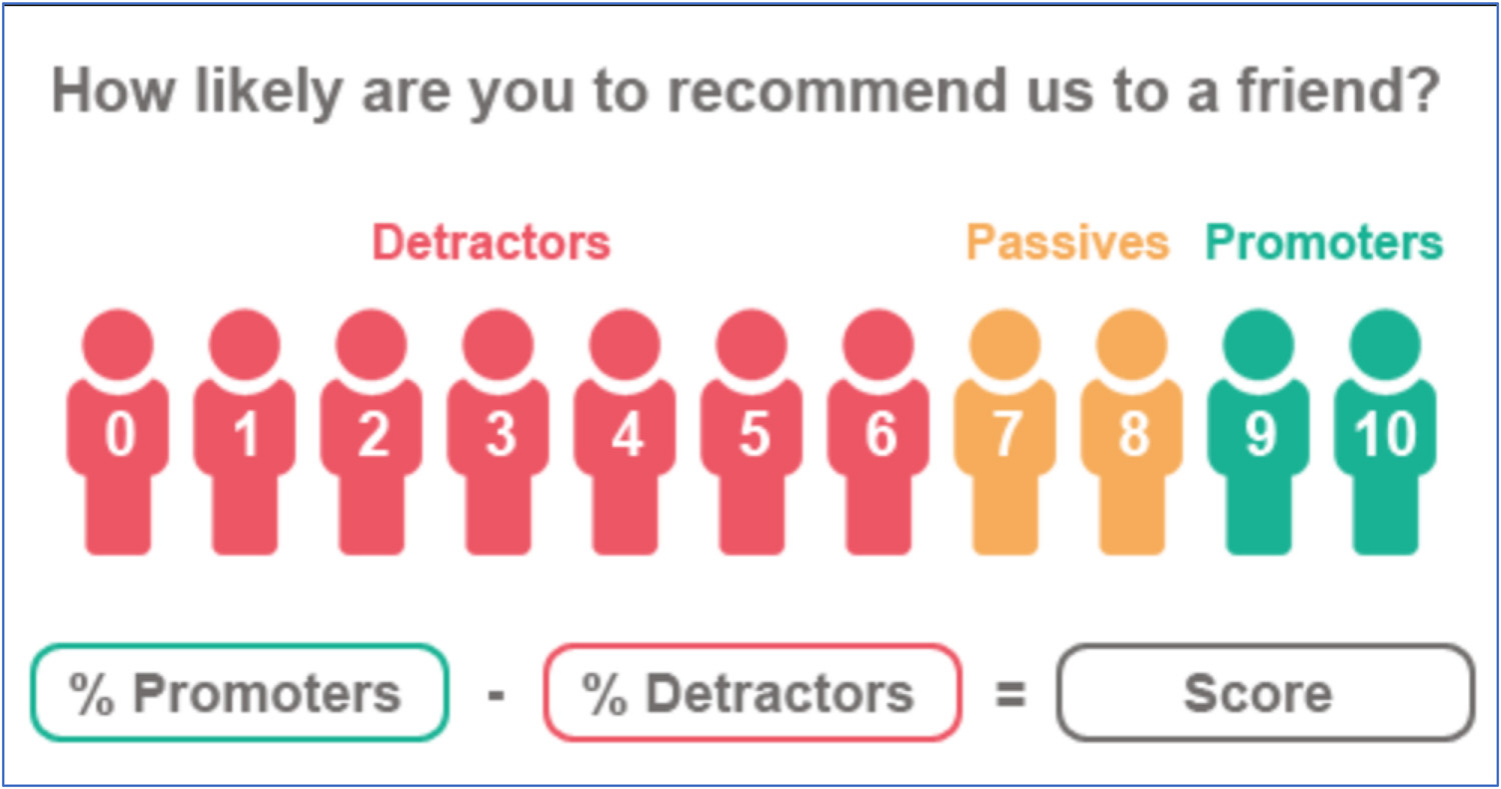
Calculating the Net Promoter Score Source: How Likely: https://www.howlikely.com/resources/nps-what-exactly-is-it

The NPS is calculated by first segmenting customers into ‘promoters’ (those rating their willingness to recommend as a 9 or 10), ‘passives’ (those rating their willingness as a 7 or 8), and ‘detractors’ (a willingness to recommend of 6 or below) and then subtracting the proportion of customers that are detractors from the proportion that are promoters, resulting in an NPS ranging from −100 to 100 (the final score is shown as an integer, not a percentage) (Figure 1).

Research showing that this simple metric is a stronger predictor of growth than more complicated and expensive customer satisfaction measures has led to widespread use of the NPS by global companies.^5^ Although not without its critics,^6^ the NPS is widely used due to its simplicity, ease of implementation, and potential for benchmarking within an industry, against competitors, and internally across products, sites, and time. As shown by the emerging body of evidence, social sector organizations have begun to incorporate the NPS into their research—testing how to adapt and use it in nonprofit settings.^7^^–^^13^

However, within the social sector, questions remain regarding the degree to which a measure developed for the profit sector is applicable in nonprofit settings. Within the context of international SRH health care and family planning services, IPPF questioned whether the NPS could be adapted for use in low-resource and low-literacy health care settings across different cultural contexts; whether it would generate meaningful data to inform service improvements; whether generating the data would streamline the data collection burden or require significant additional resources; and whether the methodology would be acceptable to clients and staff.

## METHODOLOGY

In order to make the NPS applicable for use in a health care setting, the NPS question was revised to “How likely are you to recommend this clinic to someone who needs similar services to those you received today?” The underlined section was added to address the potential that someone might hesitate to recommend the services—not because of a bad experience but rather because they would not want a friend or colleague to know they accessed SRH services, in general, or specific services such as postabortion care or HIV care and treatment.

To address the challenges of traditional client satisfaction surveys, and with the aim of finding a standardized metric that could be implemented using approaches that could be adapted for a variety of contexts, we identified opportunities within existing programmatic initiatives to iteratively test aspects of using the NPS in SRH health care clinics. Over the span of a year, we field tested the NPS as a mechanism to gather feedback about the client experience in 4 countries, using convenience samples of high-volume clinics and randomly sampling family planning or SRH clients as they exited services (Table 1):

- First, to assess the feasibility and acceptability of implementation approaches in low-resource clinical settings among clients with low-literacy levels, the NPS was implemented among 188 SRH clients in 2 clinics in Mumbai, India.
- Second, to assess whether the methodology could be used to generate meaningful comparative information about the experience of different client groups, the NPS question was integrated into an existing client profile exit survey among 590 family planning clients in 9 clinics in Kenya and Nigeria.
- Third, the feasibility of a self-administered NPS survey using tablets with the questionnaire in DHIS 2 was assessed among 226 SRH health clients in 3 clinics in El Salvador.

**TABLE 1.** Overview of Net Promoter Score Iterative Testing

	India	Kenya and Nigeria	El Salvador
**Sample size**	N=188	N=590	N=226

**What was tested**	Feasibility and acceptability of implementation approaches in low-resource clinical settings among clients with low-literacy levels	Whether the methodology could be used to generate meaningful comparative information about the experience of different client groups	Feasibility of a self-administered NPS survey using tablets with an online survey in DHIS 2

**Description**	A convenience sample of 2 peri-urban clinics was selected based on client population (low literacy), client volume, proximity to reach both clinics in a single day, and willingness to participate. Female clients exiting the clinic were asked how likely they are to recommend the service. Interviewers alternated between face-to-face interviews and guiding the respondent to a drop box to circle her response in private as she exited the clinic. The survey alternated between an 11-point numerical scale and an 11-point emoji-face scale. Face-to-face interviews included an open-ended ‘why’ question. Twenty clients were contacted for follow-up via telephone.	A convenience sample of 9 service delivery sites (6 in Kenya and 3 in Nigeria) was selected based on client volume for family planning services, representation of both static and outreach clinics, and willingness to participate. The NPS question on likeliness to recommend services was inserted into an existing client profile survey. Clients were surveyed via face-to-face interviews as they exited family planning services.	A convenience sample of 3 clinics was selected based on proximity to the capital, client volume, and willingness to participate. Volunteer youth peer promoters directed clients exiting the clinic to kiosks set up with tablets that were connected to an online survey in DHIS 2. Clients chose to complete the survey by themselves on the tablet, with assistance from a youth promoter using the tablet, or by themselves using a paper-based survey.

**Variables included in NPS survey**	Consent to participate Clinic name Interviewer name Approach: ∘ Interview: 49% ∘ Drop box: 51% Scale: ∘ Emoji faces: 49% ∘ Numerical: 51% Consent to follow-up: 96% Likeliness to recommend services: mean 9.096 Why (interviews only)	Consent to participate Country: ∘ Nigeria: 44% ∘ Kenya: 56% Service delivery channel: ∘ Static: 48% ∘ Outreach: 52% Service delivery site name Interviewer name Gender: 96% female Age: mean 30.9 years Family planning method received Source of last method used Method category: ∘ Long-acting or permanent method: 35% ∘ Short-acting reversible method: 65% Family planning use profile: ∘ Adopter (first time or lapsed): 27% ∘ Provider continuer: 34% ∘ Provider changer: 39% Reason for changing provider and/or method Likeliness to recommend services: mean 8.45	Consent to participate Clinic name Administration method: ∘ Paper-based: 9% ∘ Self-administered on tablet: 42% ∘ Youth promoter-assisted on tablet: 49% Age: mean 34.5 years Gender: 89% female Type of service Likeliness to recommend services: mean 9.39 What could be improved

Abbreviations: DHIS 2, District Health Information System 2; NPS, Net Promoter Score.

### Testing the Feasibility of Using NPS Approaches Among Low-Literacy Clients

The experience in India was designed to assess:

- whether different implementation approaches elicited different responses among low-literacy clients,
- which scale types (numerical and pictorial) were appropriate for low-literacy clients and whether these scales elicited different responses,
- whether actionable feedback could be obtained from the open-ended ‘why’ question among low-literacy clients, and
- the general acceptability of the NPS methodology by staff.

**FIGURE 2:**
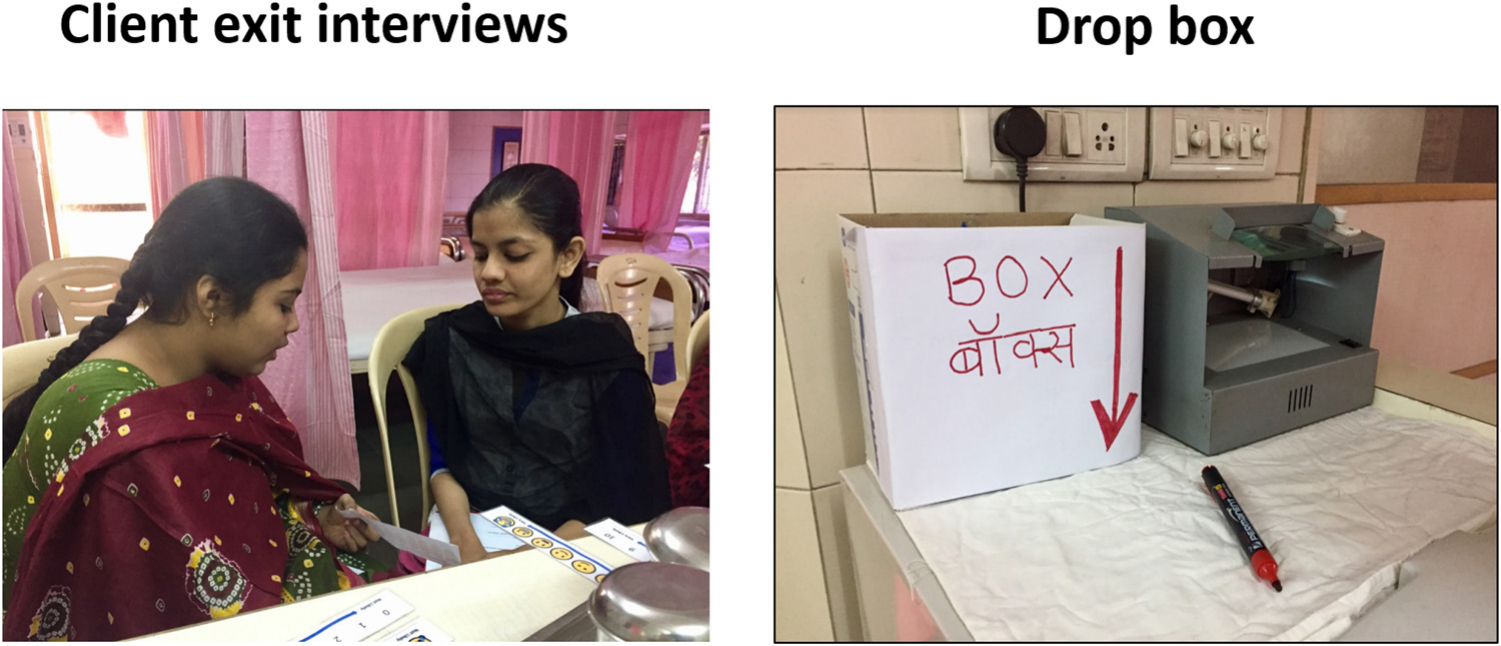
Two Net Promoter Score Implementation Approaches Tested in India

Recognizing that low-literacy clients often struggle or might be reluctant to complete a written feedback form, 2 alternative implementation approaches were tested in 2 clinics in India (Figure 2):

1. Face-to-face client exit interviews: a trained interviewer used a short, structured survey to collect the client's likelihood of recommending the clinic to someone needing similar services
2. A guided drop box approach: the interviewer explained the purpose, handed the client a strip of paper with the rating scale, and directed her to a drop box to mark in private the likeliness of recommending the clinic

Trained female interviewers systematically alternated between conducting face-to-face interviews (n=92) in Hindi or Marathi, based on client preference, and guiding clients to complete a simple form in private by circling their response on the likelihood that they would recommend the services and placing the response in a drop box on their way out of the clinic (n=96). Using the Analysis ToolPak in Microsoft Excel 2016 for all statistical analysis in our NPS study, we calculated the mean value for likeliness to recommend for the interview and for the guided drop box groups and conducted a 2-sample *t* test to determine whether the implementation approaches resulted in statistically significant differences in clients' likeliness to recommend.

Based on concerns raised by staff in India that low-literacy clients might not understand the NPS numerical scale of 0 to 10, 2 types of rating scales were also tested:

1. The standard numerical NPS scale of 0 to 10 with anchored ends (0=not at all likely and 10=very likely to recommend)
2. A face scale modeled after the Wong-Baker FACES Pain Rating Scale^14^ using an 11-point, anchored scale crafted from Microsoft emoji faces

**FIGURE 3:**
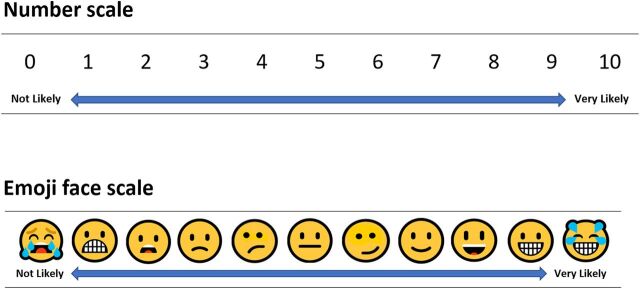
Two Types of Net Promoter Score Rating Scales Tested in India

To address the potential for different cultural interpretations of emojis,^15^ we developed the emoji face scale in conjunction with staff from our Indian partner organization. They provided changes to the initial design proposal and approved the final selection of 11 Microsoft facial emojis (Figure 3).

In addition to alternating between face-to-face interviews and the drop box approach, interviewers systematically alternated between using the numerical (n=95) or emoji scale (n=93) for clients to assess their likeliness to recommend the services. Likewise, to assess whether these 2 scales elicited different responses from clients, we calculated mean values on likeliness to recommend for all clients responding using the numerical scale and all clients using the emoji scale and conducted a 2-sample *t* test to determine whether the scales resulted in statistically significant differences in likeliness to recommend.

Finally, the pilot assessed whether actionable feedback could be obtained from the open-ended ‘why’ question typically used with the NPS using 2 approaches: asking clients the reason for their likeliness to recommend in the face-to-face interviews and asking all clients if they were willing to be contacted for follow-up and, if yes, collecting telephone details for later follow-up. Due to literacy limitations, clients using the drop box approach were not asked to respond to this question in writing.

### Assessing the Potential of the NPS to Generate Comparative Client Group Insights Through Integration of the NPS Question Into an Existing Exit Interview

To enable disaggregation and analysis by client group, the adapted NPS question on the likeliness of recommending the clinic was added at the end of an existing short exit interview designed to collect client profile data on prior family planning use among clients receiving a contraceptive method in 9 clinics in Kenya and Nigeria.

In addition to the NPS question, clients were asked a series of questions about their age, today's family planning method, and previous experience using family planning methods. This resulted in an NPS dataset that could be disaggregated by a variety of client population subgroups (Table 1).

Where relevant, client cohort binary groups were created from the sample—for example, youth clients under age 25 and adult clients age 25 and older, long-acting and short-acting method users, and family planning method insertion and removal clients. For each of the binary client cohort groups, we calculated the mean likeliness to recommend the services and conducted a 2-sample *t* test to determine whether there were significant differences in likeliness to recommend the services between the binary client cohorts.

### Assessing the Use of Tablets for Self-Administered NPS Surveys

In El Salvador, a self-administered NPS survey was tested in 3 clinics using Internet-connected tablets that were set up in small kiosks. In addition to the NPS question on the likeliness to recommend the clinic, the survey included questions about the client's age and gender and the service that brought them to the clinic. The survey also included an open-ended question asking how the client's experience could be improved in the future.

**Figure:**
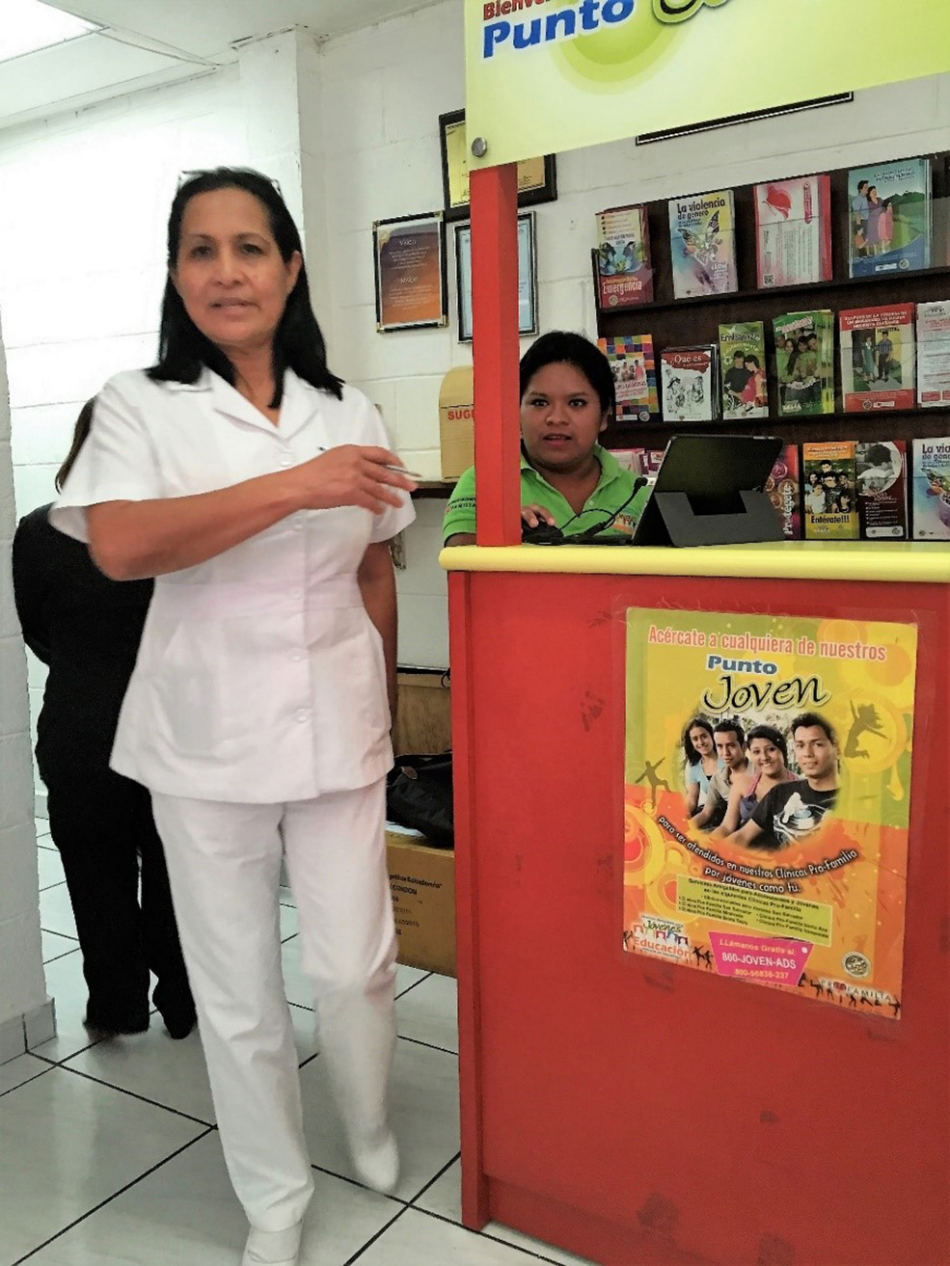
In El Salvador, a clinic supervisor and youth peer promoter tend a kiosk where the Net Promoter Score survey is set up for clients to provide feedback. © 2017 Moira Mendoza/International Planned Parenthood Federation/Western Hemisphere Region

To facilitate data collection, analysis, and warehousing, we developed an NPS survey in District Health Information System 2 (DHIS 2) software (a free and open-source health management information system used by both public and private sectors in more than 60 countries for collection, validation, analysis, and presentation of aggregate and client-based statistical data). Using an online survey eliminated the need for additional data entry, thereby reducing costs and producing real-time results.

Volunteer youth peer promoters directed SRH clients to the survey as they exited services; varying levels of literacy and comfort with technology were addressed by offering the client 3 options for the survey:

- Completing the survey themselves on the tablet
- Completing the survey on the tablet with assistance from youth promoters
- Using a paper-based survey completed by the client themselves

## FINDINGS

### NPS Implementation Approaches for Low-Literacy Clients

In India, interviewers reported that clients understood the NPS question without requiring additional explanation. As no issues were encountered with clients responding to the NPS question when they were guided by an interviewer to the drop box, the interviewers also concluded that the drop box approach was effective with low-literacy clients. However, the mean value for likeliness to recommend using the guided drop box approach was lower (*P*<.01) compared with the interview approach (Table 2). We interpreted the difference in the NPS as an indication that the drop box approach reduces courtesy bias—clients are more likely to feel comfortable giving a lower assessment when marking the response in private compared with providing the information to an interviewer.

**TABLE 2.** Two-Sample *t* Test Results Comparing Likeliness to Recommend Services of Client Groups

Client Group	No.	Mean	Standard Deviation	T-Value Calculator	T Critical Values	Degrees of Freedom	*P* Value
**India (N=188)**							
**Implementation Approach**							
Interview	92	9.52	1.09	3.11	2.61	131	.002
Drop box	96	8.69	5.75				
**Scale Type**							
Numerical scale	95	8.94	4.00	1.16	2.60	185	.25
Emoji scale	93	9.26	3.24				
**Kenya and Nigeria (N=590)**							
**Age Cohort**							
Adult clients	472	8.54	1.56	4.20	1.97	202	<.001
Youth clients	118	8.06	1.18				
**Clinic Type**							
Outreach clinics	306	8.47	1.06	0.53	1.96	513	.60
Static clinics	284	8.42	2.03				
**Method Type**							
Short-acting method	383	8.53	1.42	2.24	1.96	587	.03
Long-acting method	206	8.29	1.70				
**Insertion vs. Removal**							
IUD/implant insertion	205	8.29	1.71	2.43	1.97	178	.02
IUD/implant removal	69	7.96	0.75				
**El Salvador (N=226)**							
**Age Cohort**							
Adult clients	177	9.38	1.27	0.86	2.00	62	.40
Youth clients	48	9.58	2.25				

Abbreviation: IUD, intrauterine device.

Notes: Adult clients were ages 25 and older; youth were under age 25.

Using a drop box where low-literacy clients can respond to the NPS question in private may reduce courtesy bias.

The experience in India also demonstrated that clients with low literacy were comfortable with the traditional NPS numerical scale of 0 to 10, as there was no statistically significant difference in mean likeliness to recommend the services when using the emoji versus numerical scales (Table 2). In addition, interviewers reported that clients intuitively understood the 0-to-10 number scale while the emoji face scale required explanation; this led us to recommend using the numerical scale in future testing, even with low-literacy clients, despite the initial hesitation of staff.

Despite initial hesitation of staff, low-literacy clients understood the NPS numerical scale of 0 to 10.

### Contacting Clients for Follow-Up and Eliciting Meaningful Feedback

In India, almost all of the participating clients (96%) were willing to be contacted and provided their telephone contact details. To assess whether follow-up calls are a feasible approach for eliciting qualitative feedback, we contacted 20 clients via telephone to ask the reason for their rating; of those, we reached 19 clients (95%), indicating that contacting clients via telephone for follow-up is feasible, at least for clients in a peri-urban setting. This approach, however, may be less feasible in rural locations, where the ability to reach clients may be lower because fewer clients have mobile phones, or if the phone belongs to the client's husband rather than the client herself—an important consideration when conducting follow-up with SRH clients.

While this demonstrated that following up with clients was possible, eliciting critical feedback—that could be used for action planning to improve the service provision—using an open-ended ‘why’ question both in the face-to-face interviews and in the follow-up phone calls was challenging, even with the addition of probing questions in the phone calls. Clients offered consistent and compelling reasons why they would recommend the services—for example, the staff put them at ease and their concerns and problems were addressed at the clinic—but offered few specific suggestions on how to improve the services. Further work is needed to investigate how to elicit actionable feedback, particularly in hierarchical and deferential cultures.

Attempts to elicit critical, actionable feedback from clients using the open-ended ‘why’ question typical of Net Promoter Score surveys did not yield substantive information in India.

### The Power of Disaggregating NPS Data

The analysis from integrating the NPS question into client profile exit interviews in Kenya and Nigeria illustrated how combining the NPS question with a few targeted client demographic questions—such as age, service delivery channel, and method choice—can provide a powerful framework for identifying specific client populations who need an improved client experience.

By comparing the relative NPS of the client groups, clinic staff can quickly see which clients are less likely to recommend the services. Those client groups can then be targeted to obtain qualitative feedback about how to improve specific aspects of quality from their perspective. For example, data collected in Kenya and Nigeria showed that NPS varied across client subgroups. As explained above, to determine whether the differences among client groups was statistically significant, we calculated the mean value on likeliness to recommend the services and conducted a 2-sample *t* test. This analysis demonstrated that adult clients (ages 25 and older) were significantly more likely (*P*<.01) to recommend the services than youth clients (under age 25), and that clients coming to the clinic for an intrauterine device (IUD) or implant insertion were significantly more likely (*P*<.05) to recommend the clinic than were clients who had an IUD or implant removed (Table 2).

Comparing relative NPS data by client groups, such as age or service type, can be valuable for identifying which client groups are least likely to recommend the clinic, with follow-up actions to improve their experience.

Comparing relative NPS data across these client subgroups demonstrated that a targeted feedback process among specific client groups is needed to identify actions these clinics can take to improve the experience for younger clients and those undergoing family planning method removal procedures. Likewise, feedback from the client groups more likely to recommend the services—in this case, older clients and those undergoing family planning method insertions—could be obtained to identify positive aspects of their experience to be scaled up to improve the experience across client populations and sites. It is worth noting that while the lower likeliness to recommend among clients having their method removed may reflect dissatisfaction of the method more than dissatisfaction with the service itself, a targeted feedback process among removal clients may nonetheless provide insights into how to improve the experiences of IUD and implant clients.

Using the disaggregated NPS data to structure the qualitative feedback component may also address the challenge encountered in India with eliciting critical feedback. Rather than asking all clients an open-ended ‘why’ question, which may make them feel uncomfortable, particularly in deferential cultures where a client may fear saying anything critical about a valued service, drawing on the NPS data, clients can be asked specific questions that pertain to aspects of the experience for that client group as a whole. For example, feedback may be solicited from clients who have had an IUD removed by saying, “In this clinic, we've seen that some clients coming for an IUD removal are less likely to recommend the services than other clients. What could be done to improve the service for those clients?” Allowing participants the opportunity to provide feedback about their experience in a less personal way can deflect attention from the individual client and may increase their comfort in providing crucial information.

### Using Tablets for Self-Administered NPS Surveys

Among the 226 clients who completed the NPS survey in El Salvador, 49% elected to have a volunteer youth promoter help them complete the survey on the tablet, 42% opted to self-administer the survey on the tablet, and 9% chose to complete a paper-based survey. This indicates that a client's level of literacy or comfort with technology may limit the approaches used to self-administered surveys in these SRH clinics.

Nonetheless, offering clients the choice of completing an online survey on a tablet, either by self-administration or volunteer assistance, resulted in 91% of the responses being instantly available. The benefits of using this approach includes reducing costs of interviewing and data entry and having data available in real time—or when the tablets are able to access the Internet to automatically upload responses. Providing an online survey on tablets in the clinic can be an efficient methodology for implementing the NPS. An intriguing possibility with this approach that we did not test, but could further reduce costs while supporting low-literacy clients, is the use of an online survey with an audio feature that reads the questions aloud to respondents.

Using tablets with an NPS survey can reduce the costs and burden of data collection.

### Staff Acceptance of the NPS

Feedback indicated that the NPS approach was well received by both headquarters and clinic staff. Clinic staff appreciated that the NPS methodology was faster and easier for clients to complete than traditional client satisfaction surveys and noted that the open-ended question provided similarinformation as that gathered previously through client satisfaction surveys. Furthermore, implementing the NPS did not require additional resources, and, in fact, the use of tablets could reduce the amount of resources needed to collect and process data.

The Net Promoter Score approach was well received by headquarters and clinic staff.

Headquarters staff noted that when asking whether clients would recommend the services, using the NPS 0-to-10-point scale provided more nuanced and, therefore, more valuable information compared with surveys using a binary yes/no scale. In addition, the staff were enthusiastic about the possibility of benchmarking the NPS and having a single standard metric that could be used over time and across different sites, and they valued the simplicity of the NPS approach compared with traditional client satisfaction surveys. Encouragingly, subsequent testing not presented in this article indicated that clinic staff were particularly engaged as they watched the live results of NPS interviews conducted in a clinic in Latin America. In this case, simple dashboards were created in DHIS 2 that automatically presented the results of interviews carried out on tablets using an online version of the NPS survey in DHIS 2.

### Limitations

While using an opportunistic approach to test the NPS within existing programmatic initiatives was useful for providing evidence that the NPS could be used in low-resource clinic settings among low-literacy clients and demonstrating that implementation approaches could be adapted to address the specific contexts of each country, this iterative approach to testing did have some limitations. For example, because we were testing different aspects of the NPS at different times, we do not have a standard set of variables by which to analyze the likeliness to recommend services across all of the implementation sites. While all participating clinics provide a range of SRH services, such as family planning, maternal health, gynecology, HIV/sexually transmitted infection testing and care, and abortion-related care to the extent of legal limitations within each country, we do not have data from these pilot tests to report on variances in the NPS across standardized service types. To simplify the testing of implementation approaches for low-literacy clients in India, no data were collected related to the service for which clients had come to the clinic. In Kenya and Nigeria, due to the nature of the existing client profile survey within which the NPS question was added, only family planning clients were surveyed. In contrast, while data on service type were collected in El Salvador, 40% (n=90) of clients selected the ‘other’ category, leaving relatively small sample sizes—ranging from 2 to 47 clients—across the identified service types. Future implementation of the NPS by our organization will include a standardized set of variables to facilitate analysis, cross-site comparisons, and benchmarking.

In India, although we used an open-ended ‘why’ question in the pilot test, it did not generate specific suggestions on how to improve the services and we did not test alternative mechanisms for obtaining feedback. To quickly obtain actionable feedback without having to implement separate feedback processes for client groups more or less likely to recommend the services, additional existing approaches for gathering qualitative feedback within the NPS survey should be tested.

Ultimately, the power of using the NPS will be in determining whether it can be used as a mechanism to identify and implement actions in the clinic that lead to an improved client experience, and whether it is indeed an indicator of increased client volume. Do clinics with a higher NPS have greater volume? Do clinics with an increasing NPS see an increase in clients, indicating that word-of-mouth referrals are indeed occurring—or vice versa? While we plan to address these limitations through future implementation of the NPS, our initial pilot testing of the methodology does not provide sufficient information to address these and other crucial questions.

## LESSONS LEARNED

Throughout this process of testing approaches to implementing the NPS in SRH clinics, several lessons have been learned:

- The NPS question can be used effectively in low-resource clinic settings with low-literacy clients.
- Testing demonstrates that multiple approaches to implementing the NPS methodology are effective: face-to-face interviews, a guided drop box, self-administered or guided use of tablets, and integrating the NPS question into existing surveys. Clinic administrators can identify the best approach to use for their specific context while still providing a standardized metric for the client experience, even when implementation approaches vary across clinics.
- The NPS methodology may be most powerful in generating insights when combined with a few select client demographic questions, making it possible to compare relative NPS scores by client subgroup and identify client groups for whom the experience could be improved.
- The ability to benchmark is a desired use of the NPS methodology to support performance management between clinics, across services and client groups, and over time.
- The main value of the NPS for SRH clinics is not its standalone score, but rather its ability to identify client subgroups from which in-depth qualitative feedback should be gathered to learn how to improve the client experience for that population.
- Targeted approaches for gathering qualitative feedback will likely be more effective than using an open-ended ‘why’ question for identifying concrete actions to improve the client experience. Programs should test alternative ways of asking for information that enable clients to provide positive feedback while also drawing out areas for improvement, such as by asking 2 specific questions: “What did this clinic do well?” and “What could this clinic do better?” Additional approaches to obtaining effective client feedback, such as focus groups or targeted follow-up interviews with specific client groups, may be the best mechanism for identifying specific feedback that can be used to improve the client experience.

## CONCLUSION

Adapting the NPS for use with low-literacy clients is a promising approach to gaining insight into the client experience in SRH settings. With limited investment, the NPS can be adapted for use in low-resource and low-literacy health care clinics in different country settings. The NPS is attractive to headquarters and clinic staff because it is a simple, standardized metric that can be implemented using approaches adapted to the specific clinic context.

The NPS is a sensitive measure for identifying specific client populations in health care clinics for whom the client experience can be improved.

The NPS generated useful data to identify client groups that were less likely to recommend the services and for whom a qualitative feedback process could be used to identify actions to improve the client experience. However, for us, the open-ended ‘why’ question typically used in NPS surveys was insufficient for generating specific actionable insights in our international SRH clinics. Further research is needed to identify whether the NPS can be used for benchmarking, including for cross-country use where cultural contexts may lead to different expectations or norms around the likeliness to recommend a service or product.

Our experience indicates that the NPS should be used as part of a feedback process rather than as an absolute score. While focusing on a single absolute score may be tempting, without a robust dataset to generate meaningful benchmarks, the score itself is not particularly meaningful, as evidenced by the NPSs we saw that ranged from −4 to 100. Rather, we found that the immediate power of the NPS lies in helping clinic staff to unlock opportunities for analysis and greater understanding of the experience of specific client groups.

Based on testing the NPS in various field settings, we identified the following areas for additional exploration:

- What are the most effective and efficient approaches to gathering actionable qualitative feedback among the client subpopulations that have the lowest NPS?
- Does the NPS increase after actions are taken based on the feedback obtained from different client groups?
- With the aim of increasing access to key services among underserved populations, does the NPS correlate to service growth in SRH clinics? Is it apredictor of word-of-mouth referrals that lead to increased uptake of SRH services, and can it be used to identify service delivery models that should be scaled up? Are the NPS categorization thresholds for ‘promoters’ (likeliness to recommend services is 9 or 10), ‘passives’ (7 or 8), and ‘detractors’ (0 to 6) meaningful in relation to service growth?
- Can benchmarks be established and meaningfully used to spot trends and compare client experience across sites and countries within a global SRH organization?
- Can the NPS be effectively incorporated into broader quality assurance approaches and improvements?
- Is the NPS meaningful in contexts where access to other providers is limited or nonexistent?
- Does using the NPS impact the behavior of staff due to an awareness that clients are rating their services or because they use the NPS data to reflect on their practice and identify areas where changing behavior could lead to improved client experience?

## Notes

**En español**

**El Índice de Promotor Neto (IPN) para generar conocimiento sobre las experiencias de clientes en clínicas de salud sexual y reproductiva**

El IPN mide la probabilidad de que un cliente recomiende una organización a un amigo o colega en una escala del 0 al 10. Pruebas piloto en 4 países sugieren que el IPN puede ser utilizado exitosamente en clínicas sin fines de lucro y con poblaciones con bajo nivel de alfabetización. La combinación del IPN acompañado de algunos datos demográficos e información del servicio recibido puede servir como una herramienta poderosa para identificar poblaciones para las cuales su experiencia puede ser mejorada.

**RESUMEN**

La medida del Índice de Promotor Neto (IPN), comúnmente utilizada por las empresas de Fortune 500 para medir la experiencia del cliente, se calcula usando una escala del 0 al 10 para responder a siguiente pregunta: “¿Cuán probable es que usted recomiende [compañía X] a un amigo o colega?” A pesar del valor de esta metodología como predictor de crecimiento e indicador de la satisfacción del cliente para industrias con fines de lucro, la adopción del IPN ha sido más lenta en el sector social debido a preocupaciones sobre su aplicabilidad y aceptabilidad en entornos no comerciales, particularmente para poblaciones con bajo nivel de alfabetización. Para abordar estas inquietudes, llevamos a cabo una serie de estudios piloto a pequeña escala en El Salvador, India, Kenia y Nigeria con la finalidad de probar diferentes estrategias de implementación del IPN en clínicas de salud sexual y reproductiva—incluyendo entrevistas presenciales, buzón de respuestas con una guía para responder, integración de la pregunta del IPN en entrevistas existentes a la salida del cliente, y encuestas autoadministradas o asistidas por voluntarios en clínicas usando tabletas electrónicas—y comparar la escala tradicional numérica del 0 al 10 con una escala de caras emoji. Los resultados demostraron que el IPN puede ser adaptado efectivamente para clínicas de salud de bajos recursos y para clientes con bajo nivel de alfabetización mediante el uso de la escala numérica. No se encontraron diferencias estadísticamente significativas en la probabilidad de recomendar servicios entre el uso de la escala emoji versus la escala numérica en India; sin embargo, se encontró una diferencia estadísticamente significativa entre el buzón de respuestas y las entrevistas presenciales. Cuando se combinaron las preguntas demográficas y del uso de servicios, el IPN generó conocimiento acerca de los grupos de clientes que presentaban mayor o menor probabilidad de recomendar los servicios. Aunque la provisión de una encuesta en línea usando tabletas electrónicas puede ser una metodología eficiente para implementar el IPN, los métodos autoadministrados pueden ser limitados por el nivel de alfabetización del cliente o su comodidad con la tecnología. Para aquellas poblaciones de clientes con un IPN más bajo, recomendamos usar un proceso de retroalimentación cualitativa para promover retroalimentación crítica que permita identificar acciones que mejoren su experiencia. Nuestra experiencia con la prueba e implementación del IPN en clínicas de SSR en diversos ambientes sugiere que es una estrategia prometedora para generar conocimiento acerca de la experiencia del cliente en entornos de atención de salud sin fines de lucro.

## Notes

**Peer Reviewed**

**First Published Online:** August 2, 2018
